# Elevated blood pressure, heart rate and body temperature in mice lacking the XLαs protein of the *Gnas* locus is due to increased sympathetic tone

**DOI:** 10.1113/expphysiol.2013.073064

**Published:** 2013-06-07

**Authors:** Nicolas Nunn, Claire H Feetham, Jennifer Martin, Richard Barrett-Jolley, Antonius Plagge

**Affiliations:** Cellular and Molecular Physiology, Institute of Translational Medicine, University of LiverpoolLiverpool, UK; Institute of Ageing and Chronic Disease, University of LiverpoolLiverpool, UK

## Abstract

**New Findings:**

- What is the central question of this study?
  Previously, we showed that *Gnasxl* knock-out mice are lean and hypermetabolic, with increased sympathetic stimulation of adipose tissue. Do these mice also display elevated sympathetic cardiovascular tone? Is the brain glucagon-like peptide-1 system involved?
- What is the main finding and its importance?
  *Gnasxl* knock-outs have increased blood pressure, heart rate and body temperature. Heart rate variability analysis suggests an elevated sympathetic tone. The sympatholytic reserpine had stronger effects on blood pressure, heart rate and heart rate variability in knock-out compared with wild-type mice. Stimulation of the glucagon-like peptide-1 system inhibited parasympathetic tone to a similar extent in both genotypes, with a stronger associated increase in heart rate in knock-outs. Deficiency of *Gnasxl* increases sympathetic cardiovascular tone.

Imbalances of energy homeostasis are often associated with cardiovascular complications. Previous work has shown that *Gnasxl*-deficient mice have a lean and hypermetabolic phenotype, with increased sympathetic stimulation of adipose tissue. The *Gnasxl* transcript from the imprinted *Gnas* locus encodes the trimeric G-protein subunit XLαs, which is expressed in brain regions that regulate energy homeostasis and sympathetic nervous system (SNS) activity. To determine whether *Gnasxl* knock-out (KO) mice display additional SNS-related phenotypes, we have now investigated the cardiovascular system. The *Gnasxl* KO mice were ∼20 mmHg hypertensive in comparison to wild-type (WT) littermates (*P*≤ 0.05) and hypersensitive to the sympatholytic drug reserpine. Using telemetry, we detected an increased waking heart rate in conscious KOs (630 ± 10 *versus* 584 ± 12 beats min^−1^, KO *versus* WT, *P*≤ 0.05). Body temperature was also elevated (38.1 ± 0.3 *versus* 36.9 ± 0.4°C, KO *versus* WT, *P*≤ 0.05). To investigate autonomic nervous system influences, we used heart rate variability analyses. We empirically defined frequency power bands using atropine and reserpine and verified high-frequency (HF) power and low-frequency (LF) LF/HF power ratio to be indicators of parasympathetic and sympathetic activity, respectively. The LF/HF power ratio was greater in KOs and more sensitive to reserpine than in WTs, consistent with elevated SNS activity. In contrast, atropine and exendin-4, a centrally acting agonist of the glucagon-like peptide-1 receptor, which influences cardiovascular physiology and metabolism, reduced HF power equally in both genotypes. This was associated with a greater increase in heart rate in KOs. Mild stress had a blunted effect on the LF/HF ratio in KOs consistent with elevated basal sympathetic activity. We conclude that XLαs is required for the inhibition of sympathetic outflow towards cardiovascular and metabolically relevant tissues.

It has been increasingly recognized over recent years that disorders of energy balance and metabolism are often associated with cardiovascular disease. Typically, these symptoms occur jointly when central effects are involved. Specifically, the sympathetic nervous system (SNS) has been recognized as a major regulator of metabolic rate and cardiovascular physiology (Matsumura *et al.* 2003; Hall *et al.* 2010; Malpas, 2010). Obesity-related cardiovascular symptoms involve central actions of the adipokine leptin, its downstream effector, the melanocortin system, and subsequently increased sympathetic nerve activity (Hall *et al.* 2010). Much less is known about cardiovascular complications in disorders of hypermetabolism and leanness. Genetic manipulations of the renin–angiotensin system have been shown to increase metabolic rate and affect blood pressure (BP) through central as well as peripheral mechanisms (Dupont & Brouwers, 2010; Grobe *et al.* 2010). Another model with elevated sympathetic and cardiovascular tone is the Schlager inbred mouse line, which also displays reduced body weight (Davern *et al.* 2009). However, the underlying genetic cause for their phenotype is unknown. The anatomical organization of brain regions involved in the control of sympathetic activity is well known, although any differential topographical regulation of SNS outflow towards distinct peripheral tissues is less clear (Sved *et al.* 2001; Malpas, 2010; Nunn *et al.* 2011). Retrograde tracing studies from various peripheral tissues have indicated a common set of hierarchically organized brainstem and hypothalamic areas to be involved in the control of SNS outflow to diverse organs (Sved *et al.* 2001).

In this study, we analyse mice deficient for XLαs, a G-protein α-subunit encoded by the *Gnasxl* transcript of the complex *Gnas* locus (Plagge *et al.* 2008). *Gnasxl* constitutes an alternative splice variant of *Gnas* and differs only in the NH_2_-terminal exon (Fig. S1). Like G_s_α, XLαs links a number of G-protein-coupled receptors *in vitro*, which results in activation of adenylate cyclase and production of cAMP (Liu *et al.* 2011). However, the specific receptor(s) that signal through XLαs *in vivo* remain unknown. Gene expression at the *Gnas* locus is regulated by the epigenetic mechanism of ‘genomic imprinting’, which leads to the silencing of one allele depending on its parental origin (Plagge *et al.* 2008). Thus, *Gnasxl* expression occurs exclusively from the paternal allele. By contrast, *Gnas* remains expressed biallelically in most cells, with the exception of some tissues where its expression is restricted to the maternal allele, e.g. in the paraventricular nucleus (PVN) of the hypothalamus (Fig. S1; Chen *et al.* 2009). Loss of XLαs in mice results in a lean and hypermetabolic phenotype, caused by increased sympathetic stimulation of brown and white adipose tissues, and it has been proposed that there may be a systemic increase in SNS outflow (Xie *et al.* 2006). Expression of XLαs in adult mice is limited almost exclusively to the brain and correlates with SNS control centres, including the intermediolateral layer (IML) of the spinal cord, the ventrolateral medulla, medullary raphe nuclei, the nucleus tractus solitarii (NTS), hypothalamic nuclei (PVN, dorsomedial nucleus, lateral hypothalamic area, arcuate and suprachiasmatic nuclei) and the preoptic area (Pasolli & Huttner, 2001; Krechowec *et al.* 2012). Its expression in regions such as the PVN and dorsomedial nucleus suggests that it may influence wider sympathetic outflow, including targets within the cardiovascular system (Coote, 2007; Womack *et al.* 2007).

Here, we explore the cardiovascular phenotype of *Gnasxl* knock-out (KO) mice and its regulation by the autonomic nervous system. The effects of inhibition of the sympathetic and parasympathetic nervous system (PNS), respectively, were examined using reserpine, which blocks the vesicular monoamine transporter at synapses, and atropine, which inhibits muscarinic acetylcholine receptors (Janssen *et al.* 2000; Young & Davisson, 2011). To begin an investigation into potentially deregulated neuropeptide systems that might be involved in the *Gnasxl* KO phenotype, we explored responses to activation of the brain glucagon-like peptide-1 (GLP-1) system. Glucagon-like peptide-1, apart from its peripheral role, also acts as a neuropeptide produced in the NTS of the medulla (Llewellyn-Smith *et al.* 2011). As the GLP-1 receptor signals via an α-stimulatory G-protein and cAMP, this pathway could potentially be affected in XLαs KO mice. Activation of the central GLP-1 receptor has dual autonomic effects. It inhibits parasympathetic control of the cardiovascular system, resulting in increased heart rate (HR) and BP (Barragan *et al.* 1999; Yamamoto *et al.* 2002; Hayes *et al.* 2008; Griffioen *et al.* 2011), while it also stimulates sympathetic outflow towards metabolically relevant tissues (Nogueiras *et al.* 2009). Furthermore, the c-fos response to the comparatively stable GLP-1 receptor agonist exendin-4 (Ex-4), which elicits identical central effects independent of its route of injection (intraperitoneal or intracerebroventricular), coincides with brain regions that control sympathetic outflow in the hypothalamus, medulla and spinal cord (Yamamoto *et al.* 2002; Baraboi *et al.* 2011).

In this study, we present BP and HR data obtained from anaesthetized and conscious mice, respectively. Autonomic influences on HR were examined by heart rate variability (HRV) analyses. Heart rate and HRV responses to reserpine, atropine, Ex-4 and handling stress were measured. Additionally, neuronal activation in response to Ex-4 was quantified histologically via c-fos expression analysis in wild-type (WT) and KO mice.

## Methods

### Ethical approval

All animal work was approved by the Ethical Review Committee of the University of Liverpool and carried out in accordance with the UK Animals (Scientific Procedures) Act 1986 (UK Home Office Project Licences PPL40/3009 and PPL40/3351). All surgery was performed under general anaesthesia as described in detail below.

### Animals

*Gnasxl* mutant mice (Plagge *et al.* 2004) were maintained on a CD1 background. *Gnasxl* KO offspring (*Gnasxl*^m+/p−^, i.e. lacking XLαs expression from the paternally inherited allele) were produced by mating CD1 females with *Gnasxl* mutation-carrying males. All experiments were performed on young adult (∼4-month-old) male *Gnasxl* KO and WT siblings. Mice were maintained in the animal facility of the University of Liverpool on a 12 h–12 h light–dark cycle and had unlimited access to water and standard chow diet.

### Blood pressure recordings and body temperature measurements

For BP recordings via tail volume pressure recording (VPR) plethysmography, which measures systolic and diastolic pressure (CODA VPR system; Kent Scientific, Torrington, CT, USA), mice were lightly anaesthetized (urethane, 1.5 mg kg^−1^ i.p.; Sigma-Aldrich, UK) and kept in thermoneutral conditions (ambient temperature 30°C) throughout recording, which commonly results in lower BP values compared with conscious mice at room temperature (Swoap *et al.* 2004). A minimum of 10 readings were taken from each mouse; any values with low (<15 ml) displacement values were disregarded. For BP recordings via cannulation, mice were anaesthetized with urethane (1.4–2.2 mg kg^−1^) and α-chloralose (7–11 μg kg^−1^; Sigma-Aldrich, UK), administered i.p. in saline; doses were matched within sibling groups. Body temperature was recorded by rectal probe 3 min after injection of anaesthetic agents and prior to application of external heat. After this, temperature was continuously monitored and maintained at 37 ± 0.5°C by heat pad and infra-red lamp. Following loss of reflexes, the trachea was intubated to facilitate breathing, and the carotid artery was cannulated with stretched PP25 tubing filled with heparinized saline, connected to a pressure transducer. The raw blood pressure signal was digitized to a PC with a CED Micro1401 (CED, Cambridge, UK) using Spike2 at 5 kHz. The raw signal was also split, AC coupled and amplified between 10 and 100 times, depending on signal strength, and recorded on Spike2 at 5 kHz. Heartbeats were annotated to the amplified AC-coupled blood pressure signal using Wabp from the Physionet suite of programs (Goldberger *et al.* 2000). The numbers of animals used are indicated in the Results section.

### Telemetry surgery, recording and mild stress handling

Electrocardiogram transmitters (ETA-F20; Data Sciences International, St Paul, MN, USA) were implanted subcutaneously into adult mice under isoflurane–N_2_O gas anaesthesia. The mice were given preoperative s.c. injections of the analgesic buprenorphine (Temgesic, 1.5 mg kg^−1^; Reckitt Benckiser, Slough, UK), the antibiotic enrofloxacin (Baytril, 0.2 ml kg^−1^; Bayer AG, Leverkusen, Germany) and the anti-inflammatory meloxicam (Metacam, 100 μg kg^−1^; Boehringer Ingelheim, Ingelheim, Germany). The mice were monitored postoperatively, and were allowed at least 5 days of recovery before any further procedures were begun. This recovery period was found to be sufficient for the re-establishment of normal HR patterns (Thireau *et al.* 2008); we observed no differences compared with data from later time points. Mice were housed individually over receiver pads (Data Sciences International); ECG and locomotor activity were recorded continuously. The ECG signal was digitized to a PC with a CED Micro1401 using Spike2 at 5 kHz; locomotor activity was recorded at 100 Hz. Heart rate and locomotor activity were annotated using a custom program. Mild stress was induced by handling of the mice (restrained by holding the scruff; Meijer *et al.* 2006). Heart rate responses were evaluated between 120 and 180 min after reserpine injection, for 15 min after stress, and between 60 and 120 min after injection of atropine and Ex-4. The numbers of animals used are indicated in the Results section.

### Heart rate variability

Heart rate variability analysis was performed using the Kubios HRV program (Niskanen *et al.* 2004). For power spectrum analysis, HR was resampled at 20 Hz, and 3 min sections of clean and stable HR were analysed by fast Fourier transform using Welch’s periodogram with 50% overlapping windows of 32 s. Low-frequency (LF) and high-frequency (HF) bandings were 0.15–1.0 and 1.0–5.0 Hz, respectively (see results for further details).

**Figure 2:**
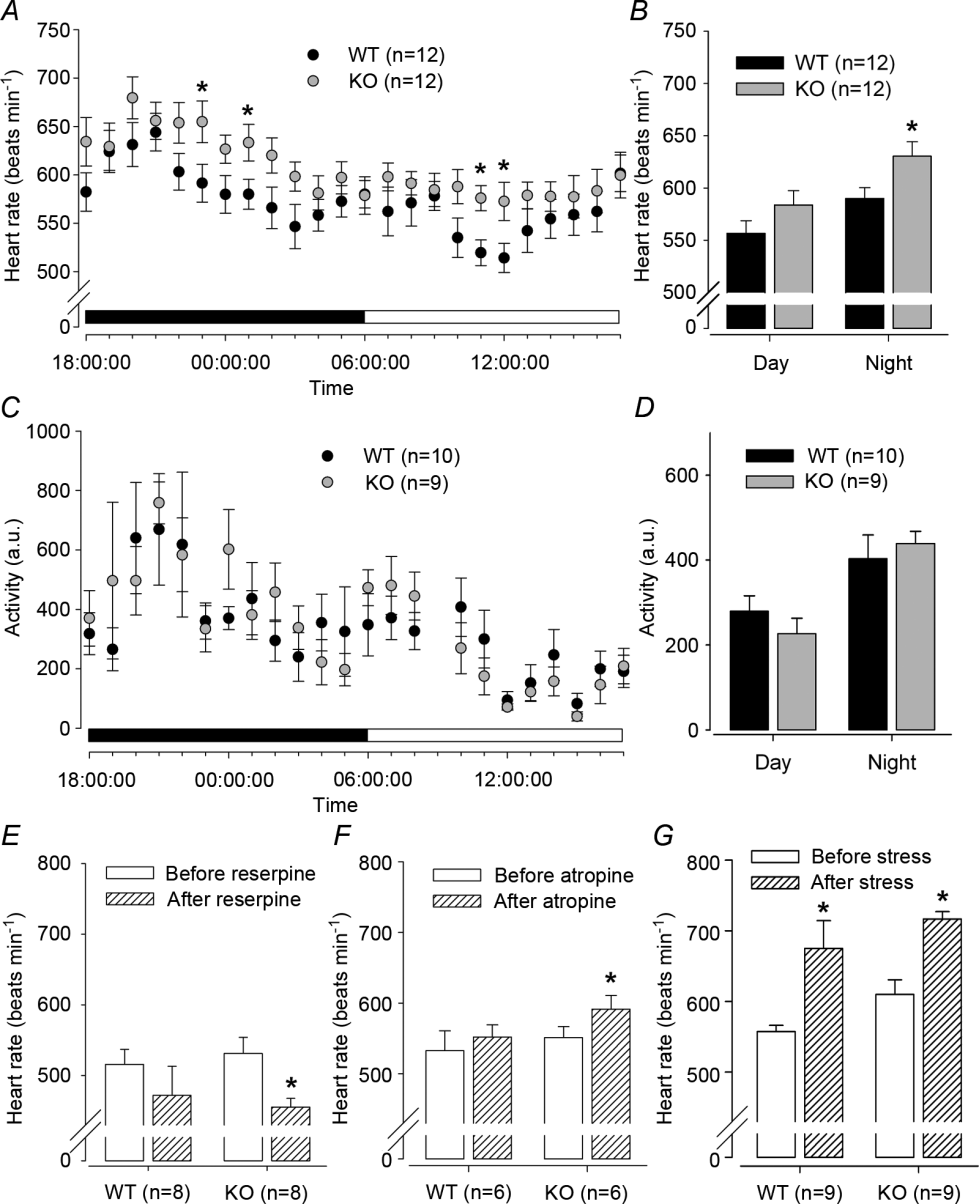
Circadian heart rate (HR), activity and HR responses in conscious *Gnasxl* KO mice Heart rate and activity were recorded continuously in freely moving KO mice and WT siblings via ECG telemetry. *A*, 1 h averaged circadian HR over 24 h. Significantly different time points are indicated (*P≤ 0.05, *n*= 12, repeated-measures ANOVA). Filled bar, night period; open bar, day period. *B*, mean HR is increased at night in KO compared with WT mice (*P≤ 0.05), but did not reach significance during the daytime. *C*, 1 h averaged circadian activity over 24 h. *D*, mean activity is unchanged in KO compared with WT mice. *E–G*, treatments and HR recordings were undertaken during the light period. *E*, following i.p. injection of 1 mg kg^−1^ of the sympatholytic reserpine the HR was significantly decreased in KO mice and trended to be lower in WT mice. *F*, HR was significantly increased in KO mice following i.p. injection of 2 mg kg^−1^ of the parasympatholytic atropine. *G*, HR was significantly increased, to a similar extent, in both genotypes following mild handling stress. *P≤ 0.05 by Student’s paired *t* test. Error bars indicate SEM.

For analysis of HRV at night, a minimum of 10 3 min sections were selected for each mouse during a 5 h period between 20.00 and 01.00 h. This period was chosen to reflect the time of highest HR and activity (see Fig. 2*A* and *C*).

### Peripheral drug injections

Reserpine (Sigma-Aldrich, UK) was dissolved in 1% acetic acid solution; it was injected at 2 mg kg^−1^ in anaesthetized mice (cannulation experiments) and at 1 mg kg^−1^ in conscious mice (telemetry experiments). Atropine (Sigma-Aldrich, UK) and Ex-4 (Tocris, UK) were dissolved in physiological saline (0.9% NaCl) and injected at doses of 2 mg kg^−1^ and 50 μg kg^−1^, respectively. All injections were performed i.p. in 50 μl volume.

### c-fos immunostaining

Exendin-4 was injected i.p. at 50 μg kg^−1^; 2 h later mice were terminally anaesthetized with Pentobarbitone (Pentoject, Animalcare, York, UK; 160 mg kg^−1^ i.p.) and perfused transcardially with 4% paraformaldehyde in PBS. Tissues were removed, dehydrated with 30% sucrose in PBS and coronal cryostat sections prepared. c-fos immunohistochemistry was performed using rabbit anti-c-fos primary antibody (Ab5, 1:50,000 dilution; Calbiochem-Merck, Feltham, UK), followed by signal amplification using the VECTASTAIN Elite ABC kit (Vector Laboratories, Peterborough, UK) with diaminobenzidine with 0.05% (w/v) NiCl_2_ substrate (Sigma-Aldrich, UK). Immunofluorescence was used to co-localize c-fos with XLαs on WT brain sections after Ex-4 stimulation. The same c-fos primary antibody (1:2000 dilution) was combined with donkey anti-rabbit Alexa 488 secondary antibody (1:1000 dilution; Life Technologies, Paisley, UK); XLαs was detected using primary goat anti-XLαs (sc-18993, 1:200 dilution; Santa Cruz, Heidelberg, Germany) and donkey anti-goat Alexa 594 secondary antibody (1:1000 dilution; Life Technologies). Tissues from five WT and five KO mice were analysed.

### Statistical analyses

For the measurements in anaesthetized dose-matched siblings, Student’s paired *t* tests were used (Minitab Ltd, Coventry, UK). Except where stated otherwise, the telemetry data were analysed by Student’s unpaired *t* tests or repeated-measures ANOVA (for circadian time points). All graphs show data as means ± SEM.

## Results

### Elevated BP in *Gnasxl* KO mice

**Figure 1:**
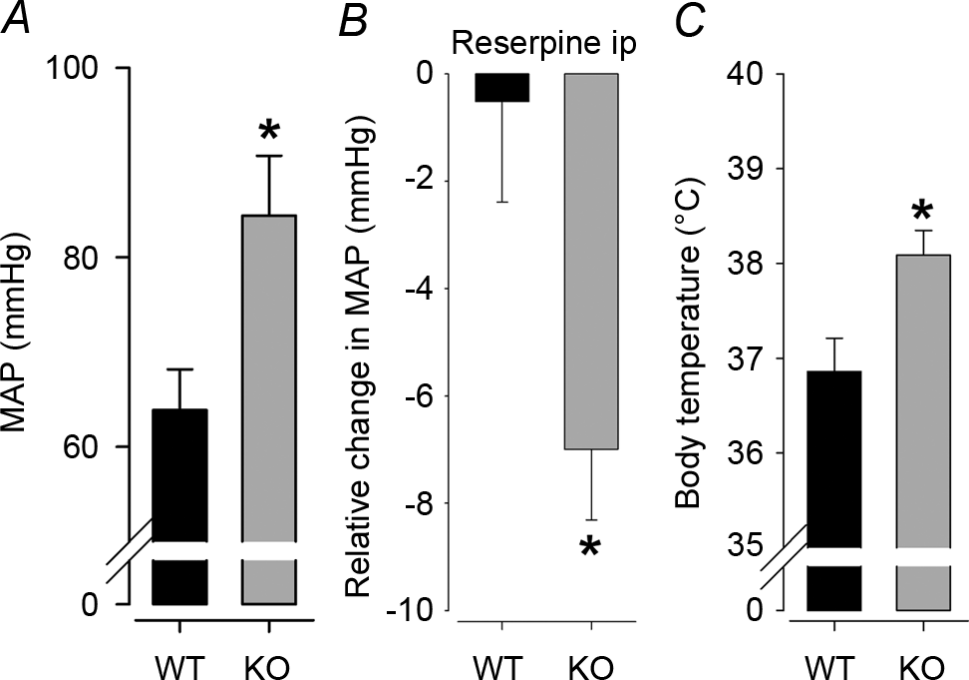
Blood pressure (BP), reserpine response and body temperature in anaesthetized *Gnasxl* knock-out (KO) mice *A*, mean basal BP (MAP) measured via tail volume pressure recording plethysmography under light general anaesthesia and thermoneutral conditions was significantly increased in KO mice compared with wild-type (WT) mice (*P≤ 0.05, *n*= 9 and 8). The systolic and diastolic data for these mice are as follows: systolic, WT 76.7 ± 5.1 mmHg *versus* KO 102.6 ± 6.4 mmHg, *P*= 0.0072; and diastolic, WT 58.3 ± 4.2 mmHg *versus* KO 74.9 ± 6.4 mmHg, *P*= 0.053. *B*, the BP change in response to reserpine was significantly greater in KO compared with WT mice, relative to vehicle, as measured via arterial cannulation (*P≤ 0.05, *n*= 5). *C*, KO mice had significantly elevated body temperature as measured via rectal probe (*P≤ 0.05, *n*= 7). Error bars indicate SEM.

Blood pressure was recorded from lightly anaesthetized male *Gnasxl* KO mice and their WT littermates via tail VPR plethysmography in thermoneutral conditions. We found mean BP to be significantly higher in KO mice than in WT siblings (Fig. 1*A*; 84 ± 6 *versus* 64 ± 4 mmHg, KO *versus* WT, *P*≤ 0.05, *n*= 9 and 8). Responses to the sympatholytic drug reserpine were recorded via arterial cannulation, to allow continuous measurement of BP. Knock-out mice had a significantly greater response to reserpine than WT mice (Fig. 1*B*; −7 ± 1 *versus*−1 ± 2 mmHg, KO *versus* WT, *P*≤ 0.05, *n*= 5). Responses to vehicle were similar in both genotypes (data not shown). These findings suggest that elevated sympathetic activity could be the underlying cause for the increased BP in mutant mice. Additionally, and in line with the previously described elevated energy expenditure (Xie *et al.* 2006), KO mice had elevated body temperature (Fig. 1*C*; 38.1 ± 0.3 *versus* 36.9 ± 0.4°C, KO *versus* WT, *P*≤ 0.05, *n*= 7).

### Increased basal HR in conscious *Gnasxl* KO mice

Heart rate data were obtained in anaesthesia-free conditions following subcutaneous implantation of telemetric transmitters. The ECG was recorded continuously in freely moving mice, and HR was derived using a custom program (see Fig. S2 for representative telemetry data). Circadian variation in HR and locomotor activity were plotted as averages per hour over a 24 h period (Fig. 2*A* and *C*). Heart rate was found to be significantly higher in KO mice at several circadian time points (Fig. 2*A*). Mean HR over 12 h day or night periods was significantly increased in KO mice compared with WT mice during the active night period (Fig. 2*B*; 630 ± 10 *versus* 584 ± 12 beats min^−1^, KO *versus* WT, *P*≤ 0.05, *n*= 12), while only a trend was observed during daytime. However, no difference in locomotor activity between genotypes was found (Fig. 2*D*).

### Autonomic control of HR in conscious *Gnasxl* KO mice

The cause of the elevated HR in *Gnasxl* KO mice was investigated by injection of reserpine and atropine to inhibit sympathetic and parasympathetic stimulation, respectively, of the cardiovascular system (Janssen *et al.* 2000; Young & Davisson, 2011). All treatments were carried out during the daytime (light) period. Reserpine at a dose of 1 mg kg^−1^ i.p. caused a significant decrease in HR in KO mice (Fig. 2*E*; KO, from 531 ± 23 to 455 ± 12 beats min^−1^, *P*≤ 0.05, *n*= 8; and WT, from 516 ± 21 to 471 ± 41 beats min^−1^, n.s., *n*= 8; Student’s paired *t* test). Atropine at a dose of 2 mg kg^−1^ i.p. caused a significant increase in HR in KO mice (Fig. 2*F*; from 551 ± 16 to 591 ± 19 beats min^−1^, *P*≤ 0.05, Student’s paired *t* test, *n*= 6), but not in WT siblings (from 533 ± 28 to 552 ± 17 beats min^−1^, *n*= 6). These data, combined with the HRV analysis described below, which shows the effects of reserpine and atropine more clearly, suggest that increased sympathetic tone causes elevated HR in *Gnasxl*-deficient mice and that inhibition of the parasympathetic system emphasizes this effect. Additionally, we investigated the cardiovascular reactivity of KO mice to mild handling stress (Meijer *et al.* 2006). Heart rate increased to a similar degree in response to stress in both WT mice (Fig. 2*G*; from 568 ± 7 to 675 ± 39 beats min^−1^, *P*≤ 0.05, *n*= 9) and KO mice (from 615 ± 20 to 716 ± 11 beats min^−1^, *P*≤ 0.05, *n*= 9).

### Analysis of HRV

In the context of abnormal HR phenotypes, HRV analysis, especially the ratio of LF to HF HRV bandings, can provide additional information on sympathetic *versus* parasympathetic balance (Malpas, 2002; Baudrie *et al.* 2007; Laude *et al.* 2008; Thireau *et al.* 2008). We used this approach via power spectrum analysis and tested autonomic influences on the cardiovascular system in conscious mice. The LF and HF HRV bandings were validated empirically following inhibition of sympathetic and parasympathetic cardiovascular tone of WT mice with reserpine and atropine, respectively (Fig. S3). High-frequency power was then used to indicate the degree of parasympathetic stimulation of the cardiovascular system, and the LF/HF ratio was used to indicate the degree of sympathetic stimulation, specifically as a measure of sympathovagal balance (Young & Davisson, 2011).

**Figure 3:**
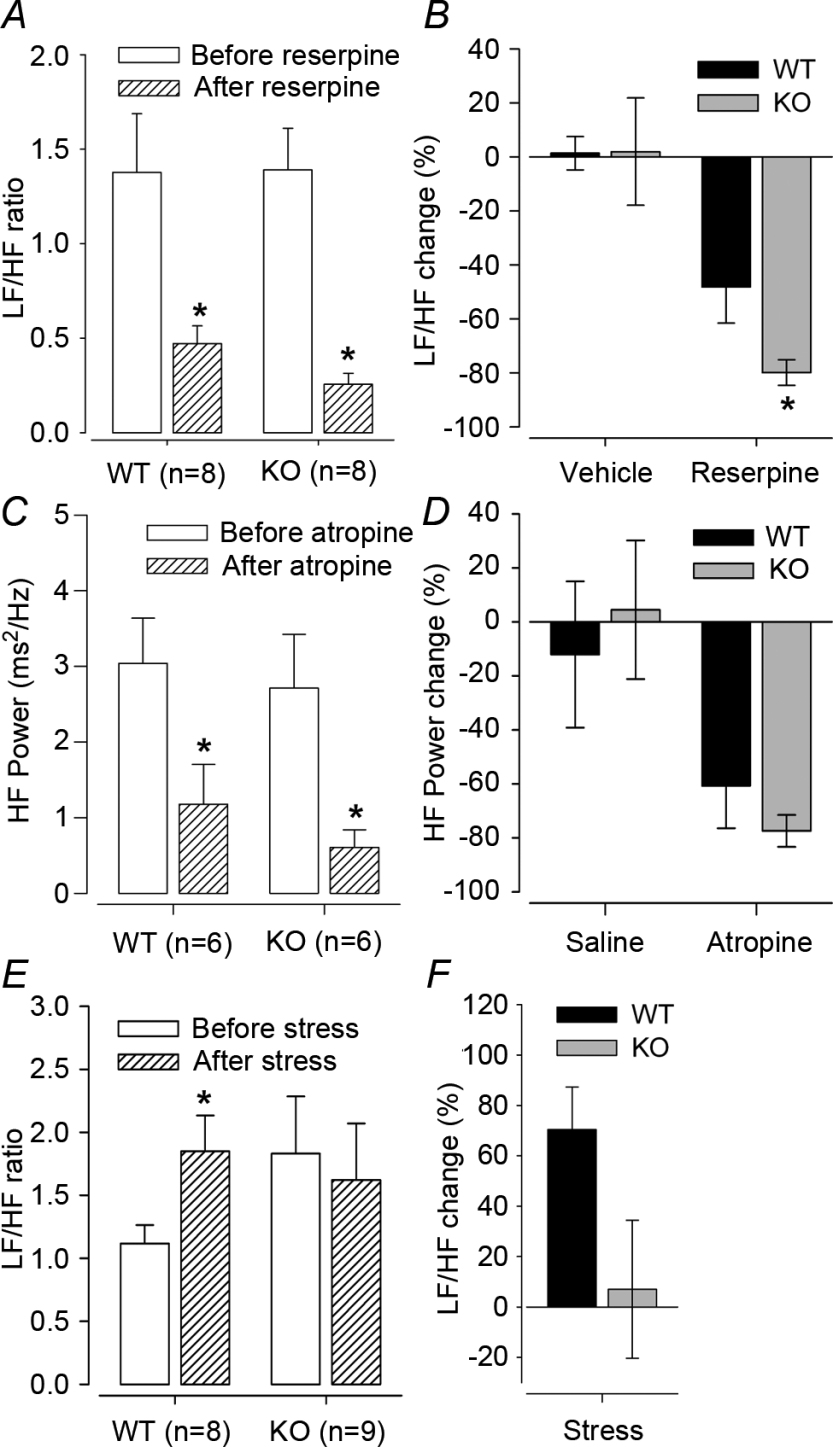
Heart rate variability (HRV) responses Treatments and HR recordings by telemetry in conscious *Gnasxl* KOs and WT siblings were carried out during the light period. Low-frequency/high-frequency (LF/HF) ratio was used as an indicator of sympathetic stimulation of the cardiovascular system, specifically of sympathovagal balance, and HF power as an indicator of parasympathetic stimulation. *A*, WT and KO mice had a significant decrease in LF/HF ratio following i.p. injection of 1 mg kg^−1^ reserpine. *B*, KO mice had a significantly greater relative decrease in LF/HF ratio compared with WT mice. *C*, both genotypes had a significant decrease in HF power following i.p. injection of 2 mg kg^−1^ atropine. *D*, both genotypes had a similar relative decrease in HF power. *E*, LF/HF ratio was significantly increased in WT mice following stress, but not in KO mice. *F*, the comparison between genotypes of relative LF/HF change following stress did not reach significance. *P≤ 0.05 by Student’s paired *t* test, or by Student’s unpaired *t* test in *B*. Error bars indicate SEM.

Autonomic control of HRV in conscious *Gnasxl* KO mice was examined by measuring LF/HF responses to reserpine, as well as HF power responses to atropine during the day (light) period. The LF/HF ratio was significantly reduced by reserpine in both WT and KO mice (Fig. 3*A*; WT, from 1.38 ± 0.31 to 0.47 ± 0.09, *P*≤ 0.05; and KO, from 1.39 ± 0.22 to 0.26 ± 0.06, *P*≤ 0.05; both by Student’s paired *t* test, *n*= 8). However, the decrease in LF/HF ratio was significantly greater in KO compared with WT mice (Fig. 3*B*; −80 ± 5 *versus*−48 ± 13%, KO *versus* WT, *P*≤ 0.05, *n*= 8). High-frequency power was significantly reduced in response to atropine in both WT and KO mice (Fig. 3*C*; WT, from 3.0 ± 0.6 to 1.2 ± 0.5 ms^2^ Hz^−1^, *P*≤ 0.05; and KO, from 2.7 ± 0.7 to 0.6 ± 0.2 ms^2^ Hz^−1^, *P*≤ 0.05; both by Student’s paired *t* test, *n*= 6). There was no difference in HF power decrease between WT and KO (Fig. 3*D*; −61 ± 16 *versus*−77 ± 6% in WT *versus* KO, *n*= 6). Finally, with regard to the stress response, mean LF/HF ratio was increased in WT (Fig. 3*E*; from 1.12 ± 0.15 to 1.85 ± 0.29, *P*≤ 0.05, *n*= 8) but not in KO mice (from 1.83 ± 0.46 to 1.62 ± 0.45), whose basal LF/HF ratio was already as high as that of the stressed WT siblings. The relative change in LF/HF ratio was short of a significant difference between both genotypes (Fig. 3*F*).

**Figure 4:**
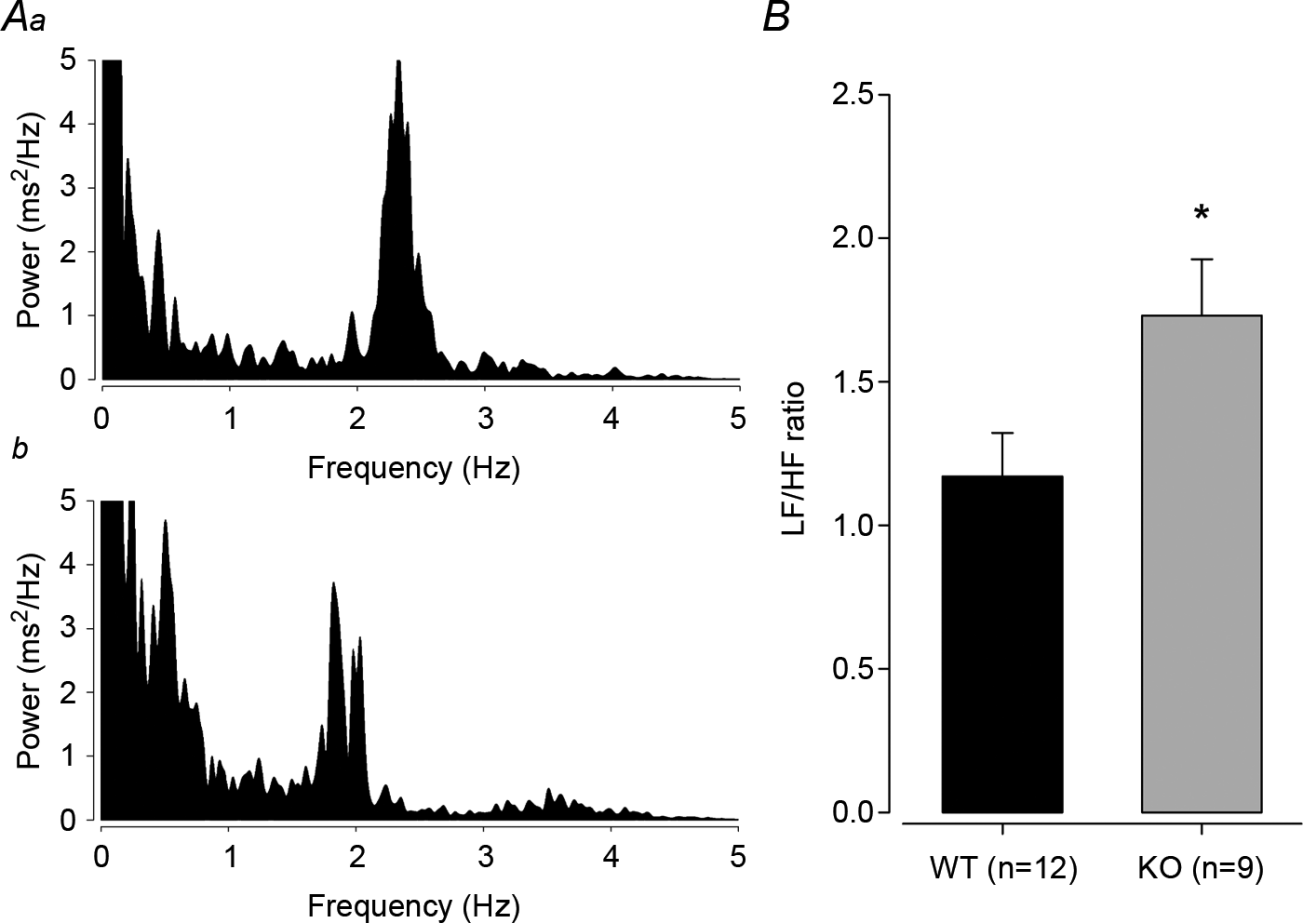
Basic HRV in conscious *Gnasxl* KO mice Heart rate spectra were produced as in Fig. 3. *A*, typical HR spectra for WT (*Aa*) and KO mice (*Ab*). *B*, LF/HF ratio was calculated for a minimum of 10 HR spectra for each mouse during the active dark period; mean LF/HF ratio was increased in KO mice. *P≤ 0.05, *n*= 9 and 12 for KO and WT, respectively. Error bars indicate SEM.

Having validated the effects of reserpine and atropine on HRV of both genotypes, we investigated basal HRV during the night phase, when HR and activity are highest. Fast Fourier transform spectra were produced from a minimum of 10 3 min windows for each mouse as discussed by Thireau *et al.* (2008; Fig. 4*A*). The *Gnasxl* KO mice had an increased LF/HF ratio at night (Fig. 4*B*; 1.79 ± 0.18 *versus* 1.23 ± 0.14, KO *versus* WT, *P*≤ 0.05, *n*= 9 and 12), which is consistent with an elevated sympathetic tone as the main cause of their cardiovascular phenotype.

### Cardiovascular responses to Ex-4

**Figure 5:**
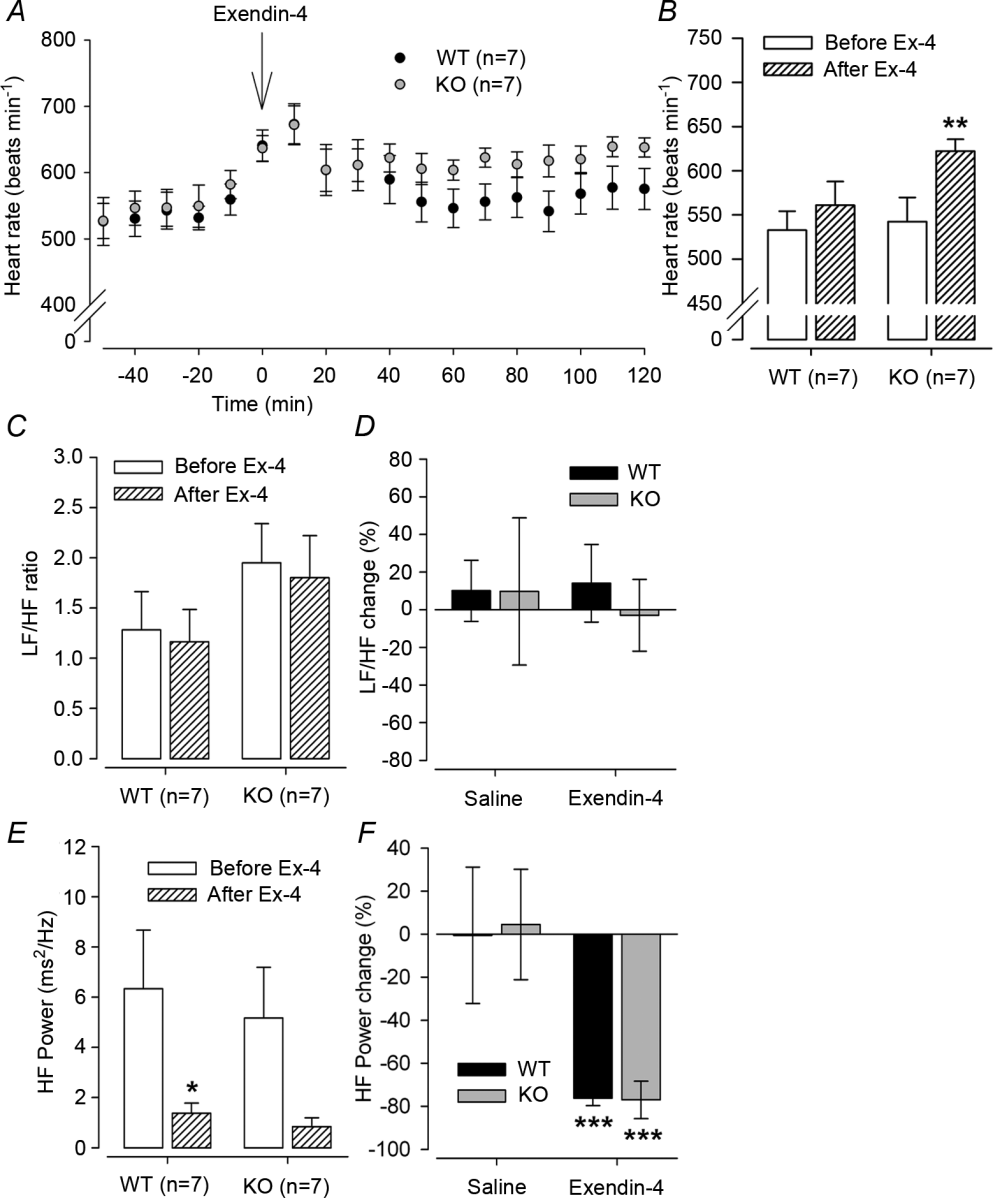
Heart rate and HRV responses to exendin-4 (Ex-4) Treatments and HR recordings were carried out during the light period as in Figs 2 and 3. *A*, HR response over time in response to i.p. injection of 50 μg kg^−1^ Ex-4 (arrow). *B*, HR is significantly increased in KO mice following Ex-4 and trended higher in WT mice. **P≤ 0.01 by Student’s paired *t* test. *C* and *D*, there was no significant difference in LF/HF ratio or relative change in LF/HF ratio following Ex-4 in either genotype. *E* and *F*, both genotypes had a decrease in HF power following Ex-4, although this reached significance only in WTs. The relative decrease in HF power was significant to a similar extent. *P≤ 0.05 and ***P≤ 0.001 by Student’s paired *t* test. Error bars indicate SEM.

Given that the GLP-1 neuropeptide system impacts on BP, HR and metabolic rate and that the GLP-1 receptor couples via an α-stimulatory G-protein, we investigated this system using the agonist Ex-4. This agonist elicits identical cardiovascular and brain c-fos responses independent of its route of injection (Barragan *et al.* 1999; Yamamoto *et al.* 2002; Hayes *et al.* 2008; Nogueiras *et al.* 2009; Baraboi *et al.* 2011; Griffioen *et al.* 2011). First, the HR response to 50 μg kg^−1^ Ex-4 was examined (injected i.p. during the daytime); it resulted in a significant increase in HR in KO mice (Fig. 5*A* and *B*; from 542 ± 27 to 622 ± 14 beats min^−1^ in KO *versus* from 533 ± 21 to 561 ± 27 beats min^−1^ in WT; *P*≤ 0.05 by Student’s paired *t* test, *n*= 7). Heart rate variability analysis indicated little effect on LF/HF ratio (sympathetic stimulation) in WT or KO mice (Fig. 5*C* and *D*). However, HF power (parasympathetic activity) was decreased significantly by Ex-4 to a similar degree in both genotypes (Fig. 5*E* and *F*; WT, −76 ± 4%; and KO, −77 ± 9%, *P*≤ 0.001; both by Student’s paired *t* test, *n*= 7), suggesting that the regulation of parasympathetic tone by the GLP-1 system (Griffioen *et al.* 2011) is functioning normally in the absence of XLαs. The effects of Ex-4 were very similar to those of the parasympatholytic atropine (Figs 2*F* and 3*C* and *D*). Both drugs caused a similar HRV (HF) response in WT and KO mice, as well as an increase in HR in KOs.

### Neuronal c-fos responses to Ex-4

**Figure 6:**
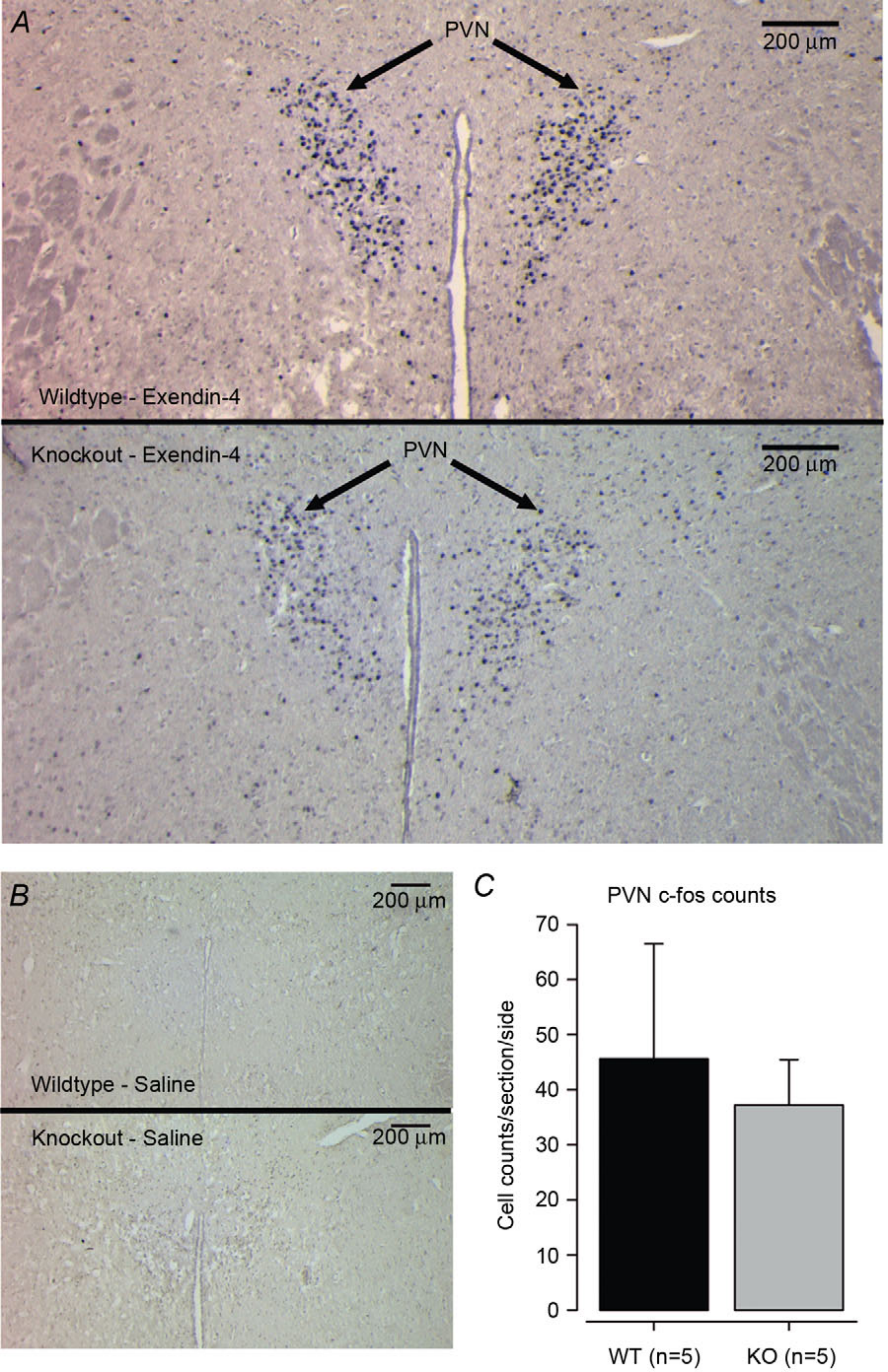
Neuronal c-fos response to Ex-4 in the hypothalamic paraventricular nucleus (PVN) c-fos immunohistochemistry of coronal brain sections after injection of 50 μg kg^−1^ i.p. Ex-4. *A*, representative images showing high levels of c-fos in the PVN of both genotypes. *B*, no significant c-fos response was observed following saline injection. *C*, there was no significant difference in numbers of c-fos-positive neurones (per section and left/right brain side) between genotypes. This was also confirmed in a more detailed anterior–posterior PVN subregion-specific assessment (data not shown). Error bars indicate SEM.

**Figure 7:**
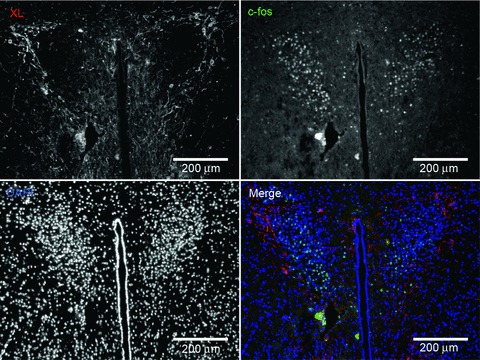
c-fos response to Ex-4 in correlation to XLαs expression in the PVN Immunofluorescence for c-fos (green) and XLαs (red) on brain sections of WT mice after Ex-4 i.p. injection. A representative image demonstrates XLαs-expressing neurones located around the periphery of the PVN, while all c-fos positive neurones are found in the centre of the PVN in a noticeably separate cell population. Of five WT mice investigated, co-localization between XLαs and c-fos was <1%.

c-fos immunohistochemistry provides a useful histological indicator of neuronal activation patterns in response to various stimuli. In contrast, neuronal inhibition does not result in c-fos induction (Dampney & Horiuchi, 2003). We investigated potential changes in the c-fos response to Ex-4 in *Gnasxl* KO mice using the same dose as in the HR experiment. Exendin-4 resulted in similar numbers of c-fos-positive neurons in the expected brain regions (PVN, NTS, area postrema and amygdala) in both genotypes (Fig. 6 and Figs S4 and S5). There was also no difference in c-fos counts when a more detailed analysis of PVN subregions along its rostral–caudal axis was undertaken (data not shown). The unchanged c-fos response in sympathetic control regions of XLαs-deficient mice further supports the view that the functions of the GLP-1 system are not impaired in KO mice. We also examined whether the Ex-4-induced c-fos response occurs in XLαs-expressing neurons in WT mice. There was no overlap of expression of the two proteins in the PVN or in the anterior part of the NTS, indicating that XLαs-positive neurons are not activated by Ex-4 (Fig. 7 and Figs S6 and S7).

## Discussion

In this study, we demonstrate, for the first time, that *Gnasxl*-deficient mice have increased BP, HR and body temperature, which is not associated with increased locomotor activity. To examine the degree of sympathetic stimulation of the cardiovascular system, mice were injected with the sympatholytic reserpine, which inhibits catecholamine uptake into secretory vesicles and thereby release at sympathetic nerve terminals (Iversen *et al.* 1965). Thus, it selectively diminishes sympathetic stimulation of peripheral tissues. Reserpine caused a greater reduction in BP, HR and LF/HF ratio of HRV in KO compared with WT mice, which concurs with the hypothesis of an increased sympathetic stimulation of the cardiovascular system. In contrast, inhibition of parasympathetic activity with the muscarinic antagonist atropine (Janssen *et al.* 2000; Laude *et al.* 2008; Young & Davisson, 2011) or the GLP-1 receptor agonist Ex-4, which was only recently discovered to affect parasympathetic cardiovascular control (Griffioen *et al.* 2011), caused similar reductions in HF power of HRV in both WT and KO mice. This indicates normal levels of parasympathetic tone in XLαs-deficient mice. The increased HR in KO mice after atropine or Ex-4 injection can be attributed to an underlying elevated basic level of sympathetic activity in mutants compared with WT mice.

For BP experiments, we chose an anaesthetic agent that affects the cardiovascular system less than other substances (Carruba *et al.* 1987). Furthermore, the anaesthetic was carefully dose matched among WT and KO littermate groups, to equalize any effect. Thermoneutral conditions, as used in our tail-cuff VPR experiment, also tend to lower mean BP (Swoap *et al.* 2004). Nevertheless, we consistently found BP and the reserpine-mediated reduction in BP to be greater in *Gnasxl* KO mice. The cardiovascular phenotype was further investigated in conscious, freely moving mice using telemetry for ECG monitoring. Knock-out mice showed an increased HR mainly during the active night period. The reserpine-mediated reduction in HR of KO mice suggests increased sympathetic stimulation of the cardiovascular system. Furthermore, a significant increase in the HR of *Gnasxl*-deficient mice was observed after application of the muscarinic blocker atropine. These findings led us further to investigate HRV data, which inform on autonomic control of the cardiovascular system and would exclude potential peripheral causes (Young & Davisson, 2011).

Heart rate variability analysis is a method for quantifying autonomic influences on the cardiovascular system based on the inherent rhythms involved in HR variation over time. These occur at characteristic frequencies associated with SNS and PNS influences. Heart rate variability is widely used as a helpful and accurate indicator of autonomic balance (Baudrie *et al.* 2007; Laude *et al.* 2008; Thireau *et al.* 2008) and autonomic responses (Farah *et al.* 2006; Griffioen *et al.* 2011). As there are no universal HRV indicators that precisely map to sympathetic activity (Malpas, 2002; Thireau *et al.* 2008), we defined these empirically by using the classical sympatholytic and parasympatholytic drugs reserpine and atropine, respectively (Iversen *et al.* 1965; Janssen *et al.* 2000; Laude *et al.* 2008; Young & Davisson, 2011). Our empirical validation led us to set our HF band between 1 and 5 Hz. This is in agreement with other analyses (Thireau *et al.* 2008). We determined that reserpine resulted in loss of virtually all LF power density down to 0.15 Hz. We therefore set our LF range between 0.15 and 1 Hz. In our verifications of drug effects on LF, HF and the LF/HF ratio, atropine decreased LF and HF but had minimal effect on the LF/HF ratio, whilst reserpine decreased LF and the LF/HF ratio, without significant change in HF power. Therefore, the LF/HF ratio appropriately indicates sympathetic tone.

Using these HRV bandings, we found that KO mice had significantly increased LF/HF ratio during the active night period. In KO mice, the LF/HF change after reserpine was significantly more pronounced than in WT mice, suggesting increased sympathetic tone. In contrast, atropine induced a similar HF power response in both genotypes, suggesting no difference in basal PNS activity. The combined HRV and HR data after atropine suppression of the PNS are consistent with elevated basal SNS activity as the prominent cause of the cardiovascular phenotype of *Gnasxl* KO mice.

Several neuropeptides have been shown to influence the central control of the autonomic nervous system and cardiovascular phenotype (Matsumura *et al.* 2003; Dupont & Brouwers, 2010; Hall *et al.* 2010). To begin an analysis of potentially deregulated neuropeptide pathways, which use α-stimulatory G-protein-coupled receptors for signal transduction, we analysed the GLP-1 system. In the CNS, GLP-1 is produced by neurons in the medulla oblongata (Llewellyn-Smith *et al.* 2011). It increases BP, HR and sympathetic outflow towards metabolically relevant tissues (Barragan *et al.* 1999; Yamamoto *et al.* 2002; Hayes *et al.* 2008; Nogueiras *et al.* 2009). We detected an exaggerated HR response to Ex-4 in *Gnasxl* KO mice. Our findings are consistent with a recent report on Ex-4-induced changes in HR and in the HF component of HRV (Griffioen *et al.* 2011). Together, these studies strongly suggest that Ex-4 increases HR by suppressing cardiac parasympathetic tone. Although Ex-4 reduced the HF component of HRV to the same degree in WT and KO mice, the increase in HR was more pronounced in XLαs-deficient mice, which we attribute to a higher underlying basal sympathetic tone.

In parallel to the suppression of parasympathetic cardiovascular tone, the GLP-1 system also affects the SNS and the hypothalamic–pituitary–adrenal axis (Gil-Lozano *et al.* 2010). Exendin-4 induces c-fos expression in neurons that project to the sympathetic preganglionic neurons of the spinal cord, as well as in corticotropin-releasing factor-positive neurons of the PVN (Yamamoto *et al.* 2002; Sarkar *et al.* 2003). It also stimulates sympathetic nerve activity to white adipose tissue (Nogueiras *et al.* 2009). A normal c-fos response and an unchanged HRV LF/HF ratio in *Gnasxl* KO mice indicate that Ex-4 did not directly increase sympathetic stimulation of the heart. It is also interesting to note that XLαs-positive neurons did not show a c-fos response to Ex-4, which concurs with previous findings that XLαs is expressed in a neuron population different from, but in proximity to, sympathetic control neurons at several levels of the CNS (Krechowec *et al.* 2012).

Glucagon-like peptide-1 is not the only example of a neuropeptide that regulates the PNS and SNS reciprocally. A recent study showed that the melanocortin peptide (α-MSH) system inhibits brainstem parasympathetic preganglionic neurons, but activates sympathetic preganglionic neurons in the spinal cord via its MC4R receptor (Sohn *et al.* 2013). Stimulation of the melanocortin system results in increased sympathetic tone and hypertension (Hall *et al.* 2010; Sohn *et al.* 2013). As the MC4R receptor signals through G_s_α, the main protein product of the *Gnas* locus, there is an interesting antithesis between the two splice variants XLαs and G_s_α (Xie *et al.* 2006; Plagge *et al.* 2008; Chen *et al.* 2009; Sohn *et al.* 2013). Knock-out mice for XLαs and G_s_α, respectively, show opposite physiological phenotypes. While XLαs deficiency is associated with leanness and hypermetabolism (Xie *et al.* 2006), lack of G_s_α expression (from the maternal allele, i.e. *Gnas*^m−/p+^) in the brain results in obesity and hypometabolism (Chen *et al.* 2009). This G_s_α-mutant phenotype resembles MC4R deficiency (Balthasar *et al.* 2005). Brain-specific *Gnas*^m−/p+^ mice are also hypotensive, with reduced HR and a global decrease in SNS activity (Chen *et al.* 2009, 2012[Bibr b7],[Bibr b6]). In contrast, we show here that *Gnasxl* KO mice are hypertensive and tachycardic due to increased sympathetic tone, which also causes elevated energy expenditure (Xie *et al.* 2006). Whether deficiency of XLαs might lead to an increased activity of the melanocortin–MC4R–G_s_α pathway remains to be tested in future studies. It is also noteworthy that XLαs and MC4R show a similar pattern of expression in some subdivisions of the hypothalamic PVN (Kishi *et al.* 2003; Liu *et al.* 2003; Krechowec *et al.* 2012). However, whether coexpression occurs or whether separate neighbouring neuron populations are involved remains to be clarified.

The *Gnasxl* KO phenotype is also consistent with a permanently activated stress reaction (Grippo *et al.* 2003; Farah *et al.* 2006). We found that WT and KO mice had comparable HR increases in response to stress, but the increase in the LF/HF ratio of HRV seen in WT mice was absent in KO mice. This would be consistent with a model whereby the classic reduction in parasympathetic cardiac inhibition by stress (Farah *et al.* 2006) still occurs, but the sympathetic excitation does not increase further in KO mice due to an already elevated basal cardiovascular sympathetic tone.

In summary, our data indicate that *Gnasxl* KO mice display a systemic increase in SNS activity, being responsible for the cardiovascular as well as the metabolic phenotype described previously (Xie *et al.* 2006). Our findings imply that the *in vivo* role of XLαs involves suppression of SNS outflow. This hypothesis is further supported by the defined expression pattern of XLαs in specific SNS control regions of the hypothalamus and brainstem, including the PVN, dorsomedial nucleus, lateral hypothalamic area, arcuate nucleus, medullary raphe nuclei, NTS and spinal cord (Krechowec *et al.* 2012). XLαs might, for example, mediate signal transduction from a G-protein-coupled receptor(s) (Liu *et al.* 2011) in a population of neurons that inhibits neighbouring sympathetic control neurons. The nature of such a receptor currently remains unclear, however. Future experiments will be aimed at characterizing XLαs-expressing neurons and the mechanisms by which this protein inhibits sympathetic activity, including analyses of additional neuroactive substances known to function in XLαs-expressing brain regions, such as α-MSH, angiotensin or substance P (Womack & Barrett-Jolley, 2007; Womack *et al.* 2007; Nunn *et al.* 2011).

## Additional information

### Competing interests

None declared.

### Funding

This work was funded by the BBSRC Capacity Building Awards in Integrative Mammalian Biology (BB/E527104/1). A.P. was additionally supported by the MRC UK (ref. G0601256). C.H.F. was funded by a BBSRC strategic skills award to R.B.J.

## Supporting Information

**Figure S1. Simplified diagram of the *Gnas*locus**

The maternal and paternal alleles of the locus are indicated.XLαs and the alternatively spliced Gsα are encoded bythe *Gnasxl* and *Gnas* transcripts, respectively. Bothproteins have a unique first exon, and share common downstreamexons. XLαs is expressed from the paternal allele (blue);Gsα is expressed biallelically in most tissues, but in certain cell types its expression is limited to the maternal allele (red). The locus has been simplified to show only the two major protein products of the locus, and is not to scale.

**Figure S2. Typical ECG and HR traces in conscious*Gnasxl* KO mice and WT siblings**

*A* and *B*, ECG was recorded in conscious freelymoving adult KO mice *B* and WT siblings *A*. (i) Typicalraw ECG traces; (ii) high resolution region of ECG showingannotated heart beats (red bars). (iii) Typical traces of RRintervals and (iv) associated HR traces. (v) High resolution HRtraces showing substantial HRV over time. These bandings were usedfor all the following HRV data analyses. *A*, (i) Typical HRspectrum from a WT mouse under basal conditions; (ii) typicalspectrum following loss of sympathetic stimulation after reserpineinjection; (iii) typical spectrum following loss of parasympatheticstimulation after atropine injection. *B*, Both LF power andLF/HF ratio were significantly reduced after reserpine injection,with no significant change to HF power. *C*, Both LF and HFpowers were significantly reduced after atropine injection, with nosignificant change to LF/HF ratio. **P* ≤ 0.05 by paired*t* test. Error bars indicate SEM.

**Figure S4. Neuronal c-fos responses to Ex-4 in theamygdala**

*Gnasxl* KO mice and WT siblings were injected with 50μg/kg I.P. Ex-4 and tissues collected two hours later. Brainsections were stained for c-fos by immunohistochemistry. *A*,Representative images showing c-fos response in the amygdala ofboth genotypes. *B*, Representative images showing nosignificant c-fos response in the same region following salineinjection. *C*, There was no significant difference in numbersof c-fos positive neurones (per section and left/right brain side)between genotypes. Error bars indicate SEM.

**Figure S5. Neuronal c-fos responses to Ex-4 in the areapostrema (AP) and medial region of the NTS**

*Gnasxl* KO mice and WT siblings were injected with 50μg/kg I.P. Ex-4 and tissues collected two hours later. Brainsections were stained for c-fos by immunohistochemistry. *A*,Representative images showing c-fos response in the AP and NTS ofboth genotypes. *B*, Representative images showing nosignificant c-fos response in the same brain regions followingsaline injection. *C*, There was no significant difference innumbers of c-fos positive neurones between genotypes. Error barsindicate SEM.

**Figure S6. Control immunofluorescence for XLαs andc-fos in response to Ex-4 in the hypothalamic PVN of *Gnasxl*KO**

*Gnasxl* KO mice were injected with 50 μg/kg I.P. Ex-4and tissues collected two hours later. Brain sections wereco-stained for c-fos (green) and XLαs (red). A representativeimage is shown, demonstrating the specificity of the XLαsantibody and a similar c-fos response as in WTs.

**Figure S7. c-fos response to Ex-4 in XLαs-expressingneurones of the amygdala**

WT and KO mice were injected with 50 μg/kg I.P. Ex-4 andtissues collected two hours later. Brain sections were co-stainedfor c-fos (green) and XLαs (red). A representative image isshown, demonstrating that XLαs-expressing neurones and c-fosresponsive neurones constitute noticeably separate populations. Of5 WT mice investigated, there were no co-localised neuronesfound.

Figure S1. Simplified diagram of the Gnas locus

Figure S2. Typical ECG and HR traces in conscious Gnasxl KO mice andWT siblings

Figure S3. Empirical validation of LF and HF bandings in HRV model.

Figure S4. Neuronal c-fos responses to Ex-4 in the amygdala.

Figure S5.Neuronal c-fos responses to Ex-4 in the area postrema (AP) andmedial region of the NTS

Figure S6. Control immunofluorescence for XLαs and c-fos in response to Ex-4 in the hypothalamic PVN of *Gnasxl* KO

Figure S7. c-fos response to Ex-4 in XLαs-expressing neurones of the amygdala
