# Supplementary material for: Elevated blood pressure, heart rate and body temperature in mice lacking the XLαs protein of the Gnas locus is due to increased sympathetic tone
Source: Exp Physiol. 2013 Jun 7;98(10):1432–45. doi: 10.1113/expphysiol.2013.073064 (PMC4223506; doi:10.1113/expphysiol.2013.073064)
Supplement: Supplementary file 1 — Figure S1. Simplified diagram of the Gnas locus [file eph0098-1432-sd1.pdf]

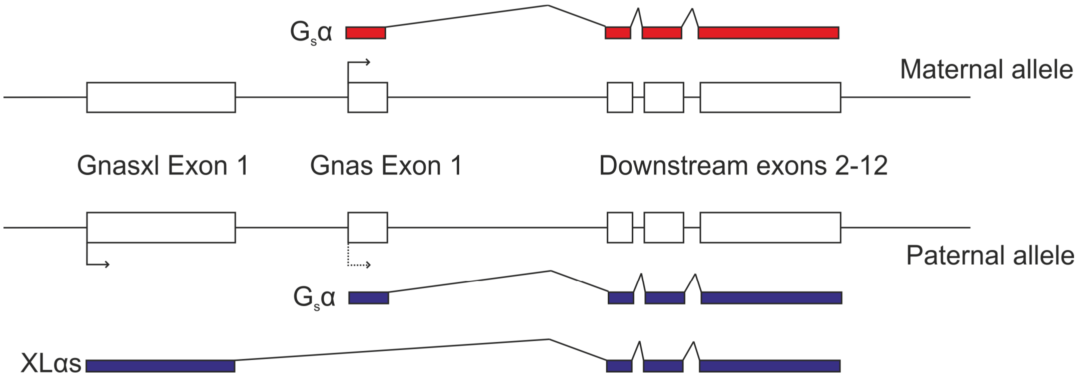

Supplemental Figure S1. Simplified diagram of the *Gnas* locus.

The maternal and paternal alleles of the locus are indicated. XLαs and the alternatively spliced $G_s\alpha$ are encoded by the *Gnasxl* and *Gnas* transcripts, respectively. Both proteins have a unique first exon, and share common downstream exons. XLαs is expressed from the paternal allele (blue); $G_s\alpha$ is expressed biallelically in most tissues, but in certain cell types its expression is limited to the maternal allele (red). The locus has been simplified to show only the two major protein products of the locus, and is not to scale.
