# Supplementary material for: Elevated blood pressure, heart rate and body temperature in mice lacking the XLαs protein of the Gnas locus is due to increased sympathetic tone
Source: Exp Physiol. 2013 Jun 7;98(10):1432–45. doi: 10.1113/expphysiol.2013.073064 (PMC4223506; doi:10.1113/expphysiol.2013.073064)
Supplement: Supplementary file 2 — Figure S2. Typical ECG and HR traces in conscious Gnasxl KO mice andWT siblings [file eph0098-1432-sd2.pdf]

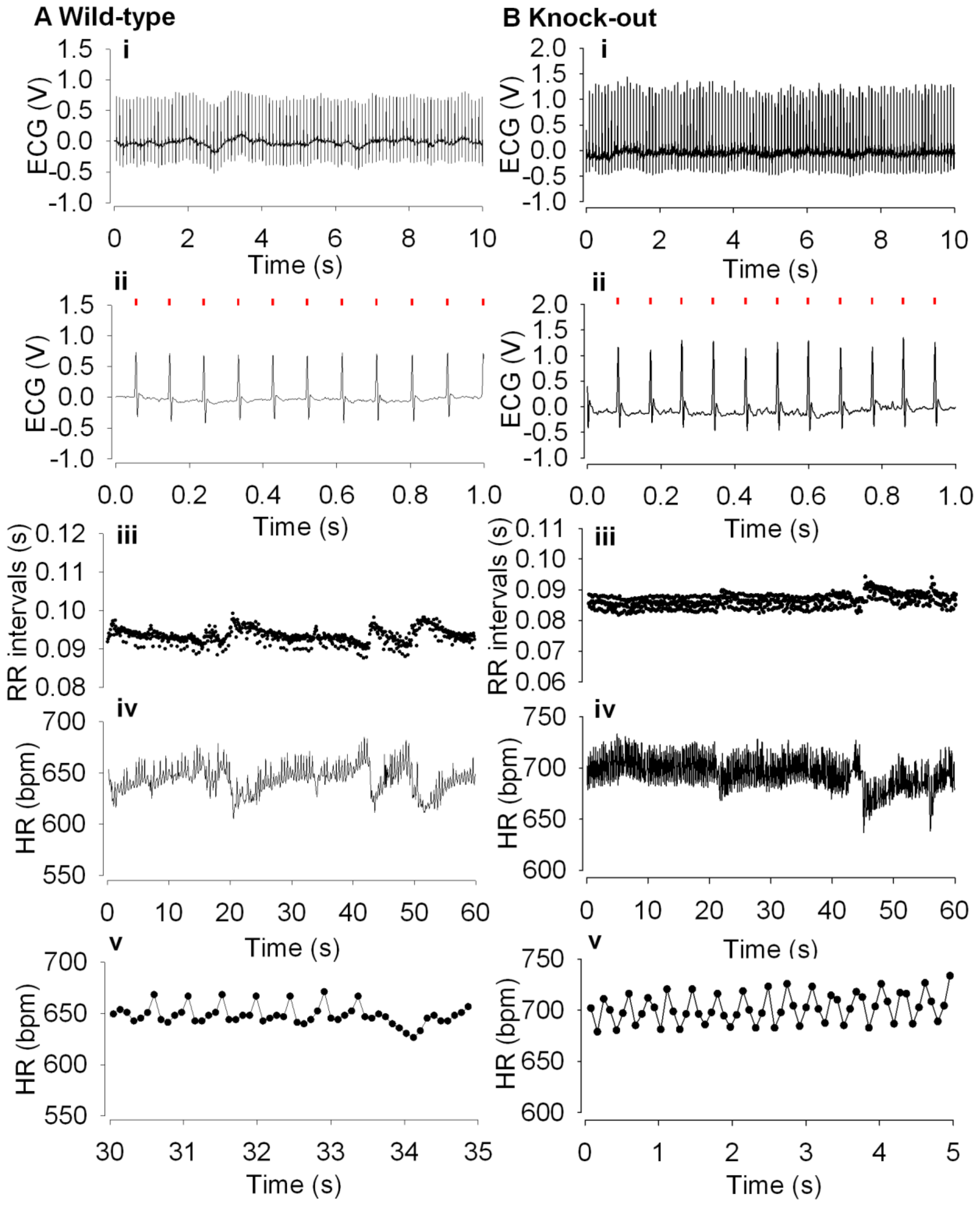

Supplemental Figure S2. Typical ECG and HR traces in conscious *Gnasxl* KO mice and WT siblings.

A and *B*, ECG was recorded in conscious freely moving adult KO mice *B* and WT siblings *A*. (i) Typical raw ECG traces; (ii) high resolution region of ECG showing annotated heart beats (red bars). (iii) Typical traces of RR intervals and (iv) associated HR traces. (v) High resolution HR traces showing substantial HRV over time.
