# Supplementary material for: Elevated blood pressure, heart rate and body temperature in mice lacking the XLαs protein of the Gnas locus is due to increased sympathetic tone
Source: Exp Physiol. 2013 Jun 7;98(10):1432–45. doi: 10.1113/expphysiol.2013.073064 (PMC4223506; doi:10.1113/expphysiol.2013.073064)
Supplement: Supplementary file 3 — Figure S3. Empirical validation of LF and HF bandings in HRV model. [file eph0098-1432-sd3.pdf]

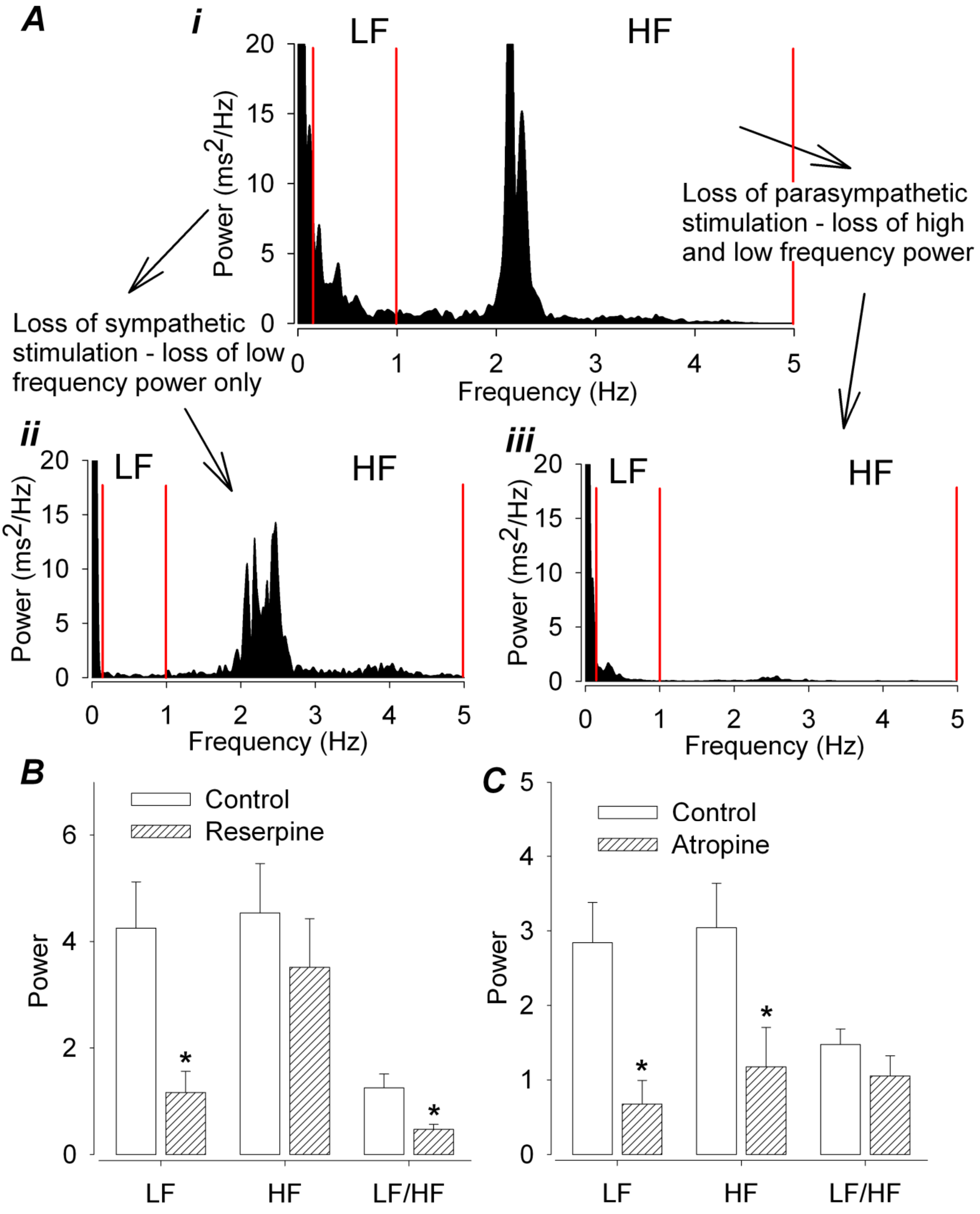

Supplemental Figure S3. Empirical validation of LF and HF bandings in HRV model. 3-minute sections of HR in conscious WT mice were analysed by FFT using Welch's periodogram with 50% overlap of 32-second windows. Reserpine was used to define the LF range, and was found to abolish all power spectral density from 0.15 Hertz (Hz) up to approximately 1.5 Hz. However, the breathing peak was occasionally found as low as 1.2 Hz, and therefore the boundary was set to 1.0 Hz to ensure no power relating to the vagal control of breathing was included in the LF region. By contrast, atropine was found to abolish virtually all power spectral density above 0.15 Hz. Therefore, the LF band was determined to be 0.15-1.0 Hz, and the HF band to be 1.0-5.0 Hz. These bandings were used for all the following HRV data analyses. *A*, (i) Typical HR spectrum

from a WT mouse under basal conditions; (ii) typical spectrum following loss of sympathetic stimulation after reserpine injection; (iii) typical spectrum following loss of parasympathetic stimulation after atropine injection. *B*, Both LF power and LF/HF ratio were significantly reduced after reserpine injection, with no significant change to HF power. *C*, Both LF and HF powers were significantly reduced after atropine injection, with no significant change to LF/HF ratio. *$p \leq 0.05$ by paired t-test. Error bars indicate S.E.M.
