# Supplementary material for: Elevated blood pressure, heart rate and body temperature in mice lacking the XLαs protein of the Gnas locus is due to increased sympathetic tone
Source: Exp Physiol. 2013 Jun 7;98(10):1432–45. doi: 10.1113/expphysiol.2013.073064 (PMC4223506; doi:10.1113/expphysiol.2013.073064)
Supplement: Supplementary file 4 — Figure S4. Neuronal c-fos responses to Ex-4 in the amygdala. [file eph0098-1432-sd4.pdf]

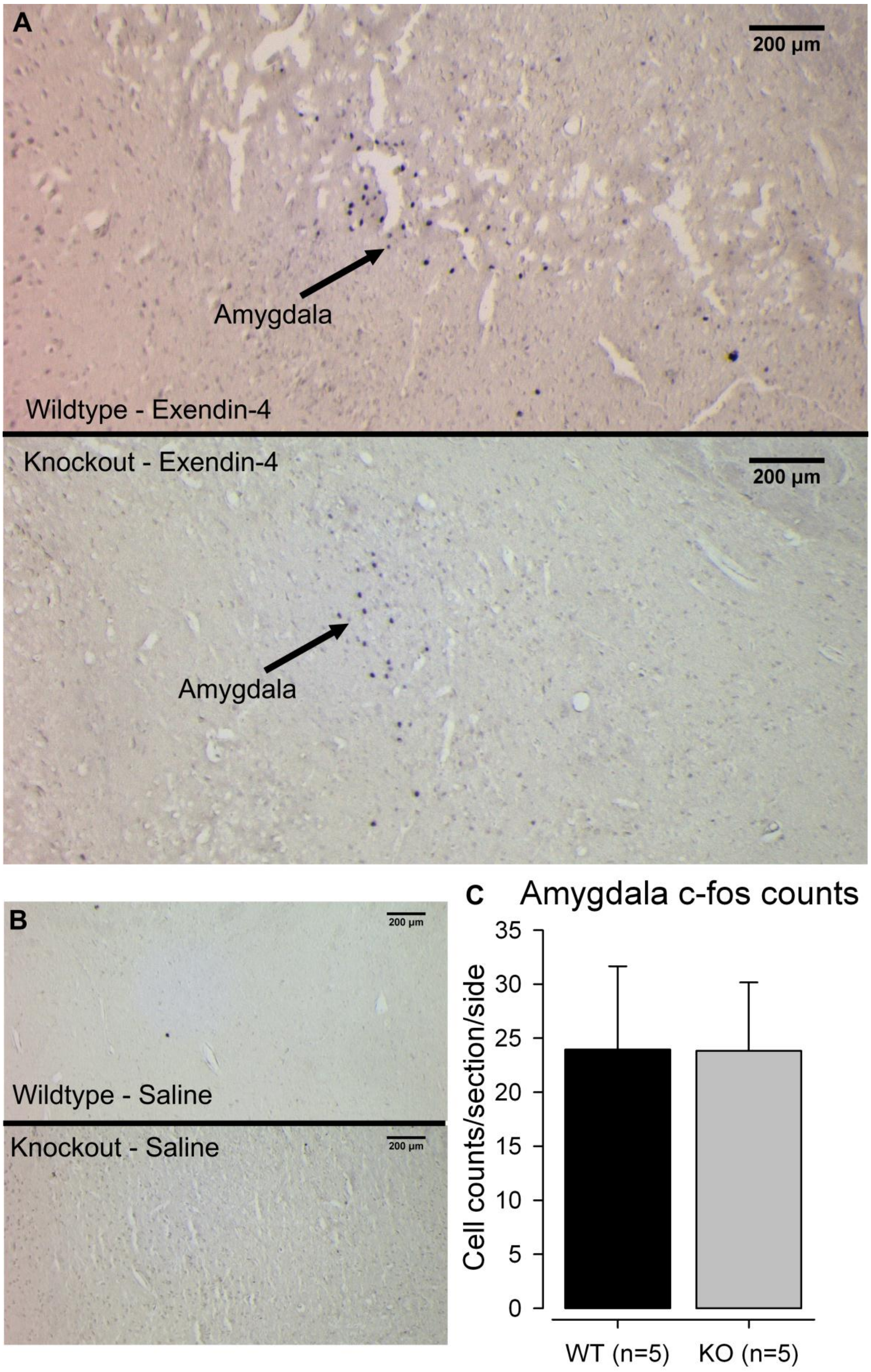

Supplemental Figure S4. Neuronal c-fos responses to Ex-4 in the amygdala.
Gnasxl KO mice and WT siblings were injected with 50 µg/kg I.P. Ex-4 and tissues collected two hours later. Brain sections were stained for c-fos by immunohistochemistry. *A*, Representative images showing c-fos response in the amygdala of both genotypes. *B*, Representative images showing no significant c-fos response in the same region following saline injection. *C*, There was no significant difference in numbers of c-fos positive neurones (per section and left/right brain side) between genotypes. Error bars indicate S.E.M.
