# Supplementary material for: Elevated blood pressure, heart rate and body temperature in mice lacking the XLαs protein of the Gnas locus is due to increased sympathetic tone
Source: Exp Physiol. 2013 Jun 7;98(10):1432–45. doi: 10.1113/expphysiol.2013.073064 (PMC4223506; doi:10.1113/expphysiol.2013.073064)
Supplement: Supplementary file 5 — Figure S5.Neuronal c-fos responses to Ex-4 in the area postrema (AP) andmedial region of the NTS [file eph0098-1432-sd5.pdf]

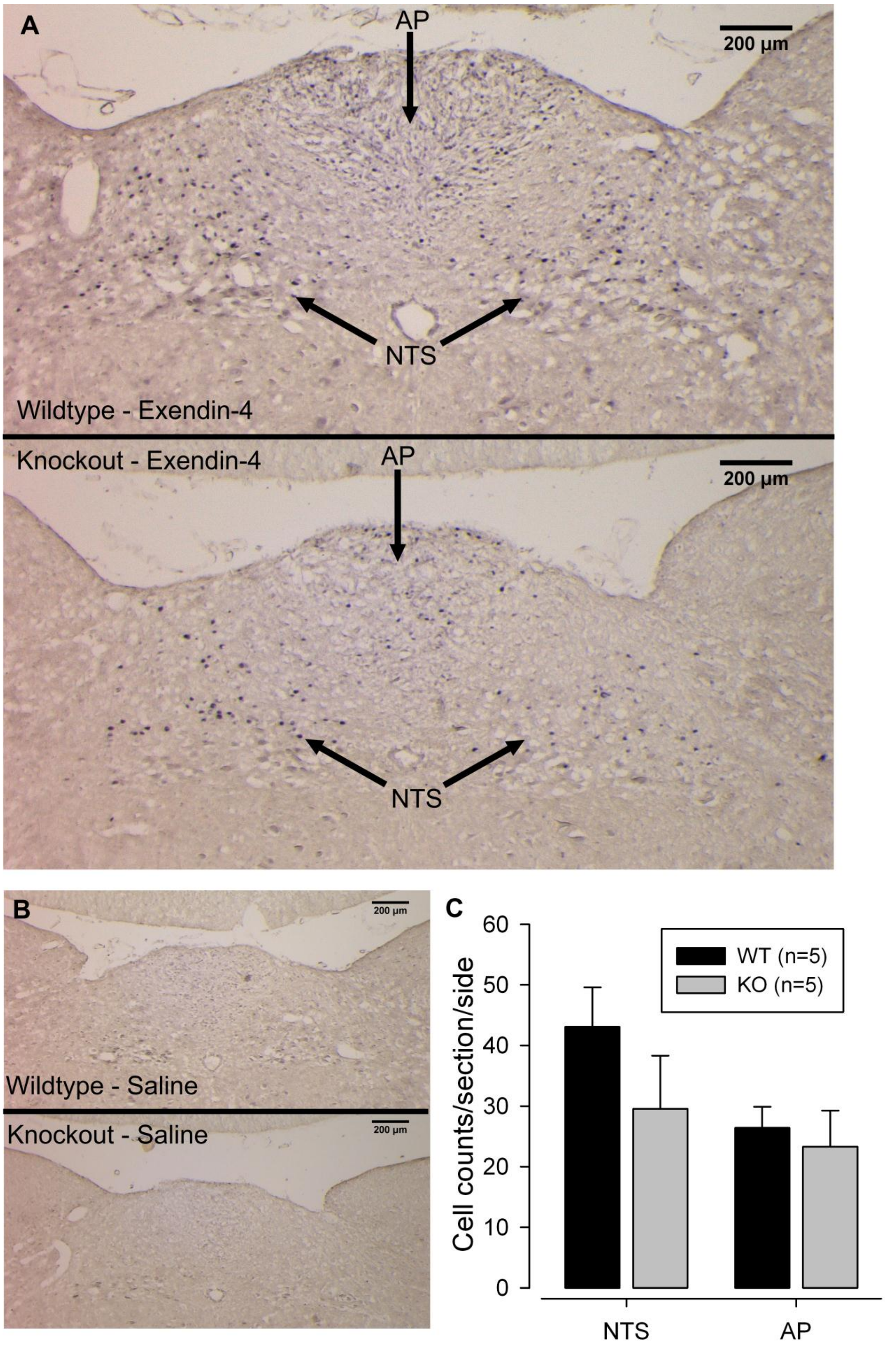

Supplemental Figure S5. Neuronal c-fos responses to Ex-4 in the area postrema (AP) and medial region of the NTS.

Gnasxl KO mice and WT siblings were injected with 50 µg/kg I.P. Ex-4 and tissues collected two hours later. Brain sections were stained for c-fos by immunohistochemistry. *A*, Representative images showing c-fos response in the AP and NTS of both genotypes. *B*, Representative images showing no significant c-fos response in the same brain regions following saline injection. *C*, There was no significant difference in numbers of c-fos positive neurones between genotypes. Error bars indicate S.E.M.
