# Supplementary material for: Elevated blood pressure, heart rate and body temperature in mice lacking the XLαs protein of the Gnas locus is due to increased sympathetic tone
Source: Exp Physiol. 2013 Jun 7;98(10):1432–45. doi: 10.1113/expphysiol.2013.073064 (PMC4223506; doi:10.1113/expphysiol.2013.073064)
Supplement: Supplementary file 6 — Figure S6. Control immunofluorescence for XLαs and c-fos in response to Ex-4 in the hypothalamic PVN of Gnasxl KO [file eph0098-1432-sd6.pdf]

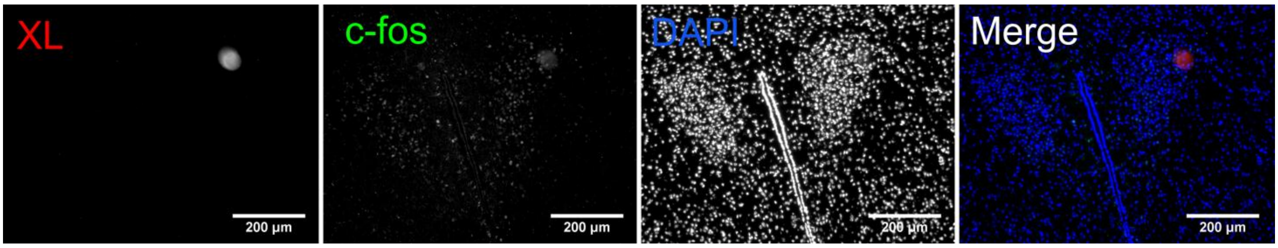

Supplemental Figure S6. Control immunofluorescence for XLαs and c-fos in response to Ex-4 in the hypothalamic PVN of *Gnasxl* KO.

Gnasxl KO mice were injected with 50 µg/kg I.P. Ex-4 and tissues collected two hours later. Brain sections were co-stained for c-fos (green) and XLαs (red). A representative image is shown, demonstrating the specificity of the XLαs antibody and a similar c-fos response as in WTs.
