# Supplementary material for: Elevated blood pressure, heart rate and body temperature in mice lacking the XLαs protein of the Gnas locus is due to increased sympathetic tone
Source: Exp Physiol. 2013 Jun 7;98(10):1432–45. doi: 10.1113/expphysiol.2013.073064 (PMC4223506; doi:10.1113/expphysiol.2013.073064)
Supplement: Supplementary file 7 — Figure S7. c-fos response to Ex-4 in XLαs-expressing neurones of the amygdala [file eph0098-1432-sd7.pdf]

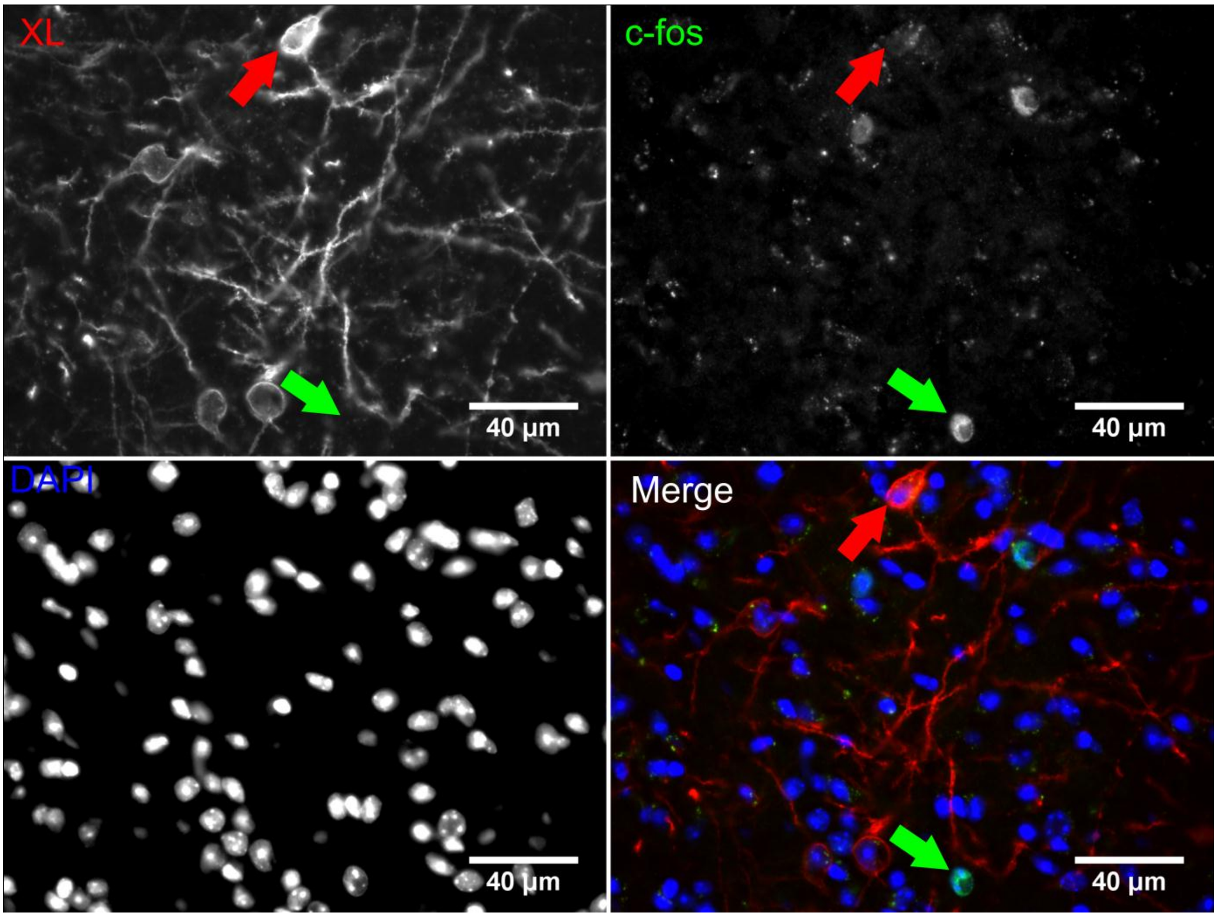

Supplemental Figure S7. c-fos response to Ex-4 in XLαs-expressing neurones of the amygdala. WT and KO mice were injected with 50 µg/kg I.P. Ex-4 and tissues collected two hours later. Brain sections were co-stained for c-fos (green) and XLαs (red). A representative image is shown, demonstrating that XLαs-expressing neurones and c-fos responsive neurones constitute noticeably separate populations. Of 5 WT mice investigated, there were no co-localised neurones found.
